# Supplementary material for: Multi-omics monitoring of drug response in rheumatoid arthritis in pursuit of molecular remission
Source: Nat Commun. 2018 Jul 16;9:2755. doi: 10.1038/s41467-018-05044-4 (PMC6048065; doi:10.1038/s41467-018-05044-4)
Supplement: Supplementary file 1 — Supplementary Information [file 41467_2018_5044_MOESM1_ESM.pdf]

Multi-omics monitoring of drug response in rheumatoid arthritis: in pursuit of molecular remission

Tasaki *et al.*

Table of Contents

1. Supplementary Figures

Supplementary Figure 1. The null distributions of the PLSR model accuracies.
Supplementary Figure 2. Correlation of the variable importance among the different data splits for the transcript-based model.
Supplementary Figure 3. Correlation of the variable importance among the different data splits for the protein-based model.
Supplementary Figure 4. Correlation of the variable importance among the different data splits for the cell-based model.
Supplementary Figure 5. RA probability distributions computed via the ensemble diagnostic models.
Supplementary Figure 6. Comparison of RA odds between training and test samples.
Supplementary Figure 7. Hierarchical clustering of gene expression profiles from 15 immune cells.
Supplementary Figure 8. Expression profiles of key transcripts in the model across 15 immune cells.
Supplementary Figure 9. Protein expression profiles of important transcripts across 26 immune cells.
Supplementary Figure 10. Protein expression profiles of key serum proteins across 26 immune cells.
Supplementary Figure 11. Correlation between treatment effects on RA odds based on three molecular classes.
Supplementary Figure 12. Biological annotations for molecular signatures affected by drug treatments.
Supplementary Figure 13. Relationship between molecular remission and individual parameters of DAS28-ESR and CDAI at 24 weeks.
Supplementary Figure 14. Relationship between the number of archived molecular remission classes and individual parameters of DAS28-ESR and CDAI in biologic-treated patients at 90 weeks during the follow-up.
Supplementary Figure 15. Correlation between disease severity indexes and transcriptional RMSs.
Supplementary Figure 16. Correlation between disease severity indexes and protein RMSs.
Supplementary Figure 17. Protein expression profiles of transcriptional RMSs across 26 immune cells.
Supplementary Figure 18. Protein expression profiles of protein RMSs across 26 immune cells.
Supplementary Figure 19. Filtering scheme for public transcriptome studies in NextBio.
Supplementary Figure 20. Comparison of fold changes for 800 untreatable transcript signatures between RA and other conditions.

2. Supplementary Tables

Supplementary Table 1. Demographics of the unmedicated cohort.
Supplementary Table 2. Demographics of 22 patients with RA receiving medication.
Supplementary Table 3. Biological concepts enriched with important variables in the protein-based model.
Supplementary Table 4. Demographics of the drug response cohort.
Supplementary Table 5. Demographics of the immune subset cohort.

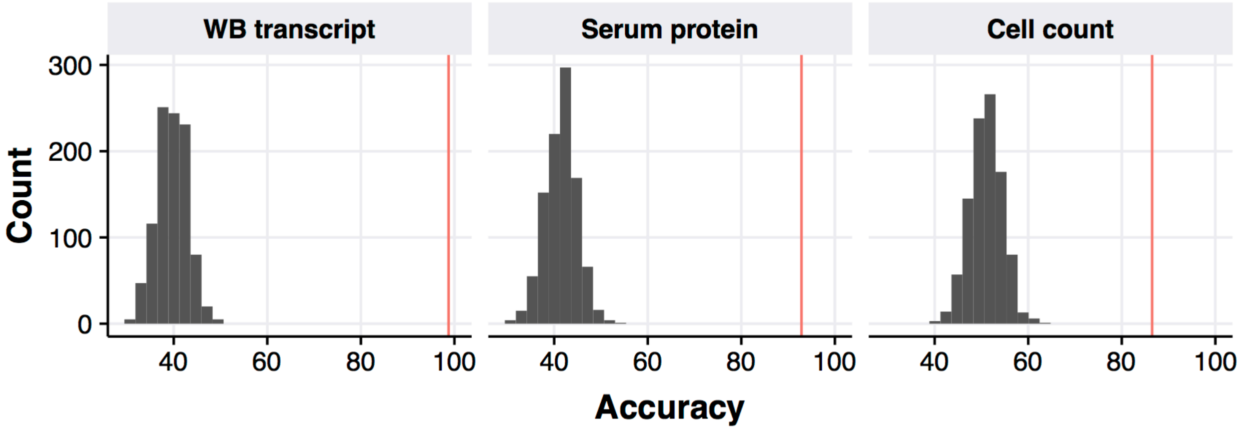

Supplementary Figure 1. The null distributions of the PLSR model accuracies. The null distributions of the averaged accuracy of 15 model ensembles for each data type were estimated via 1,000 sample label permutations. The red bars indicate the averaged accuracy of 15 model ensembles with the non-permuted data.

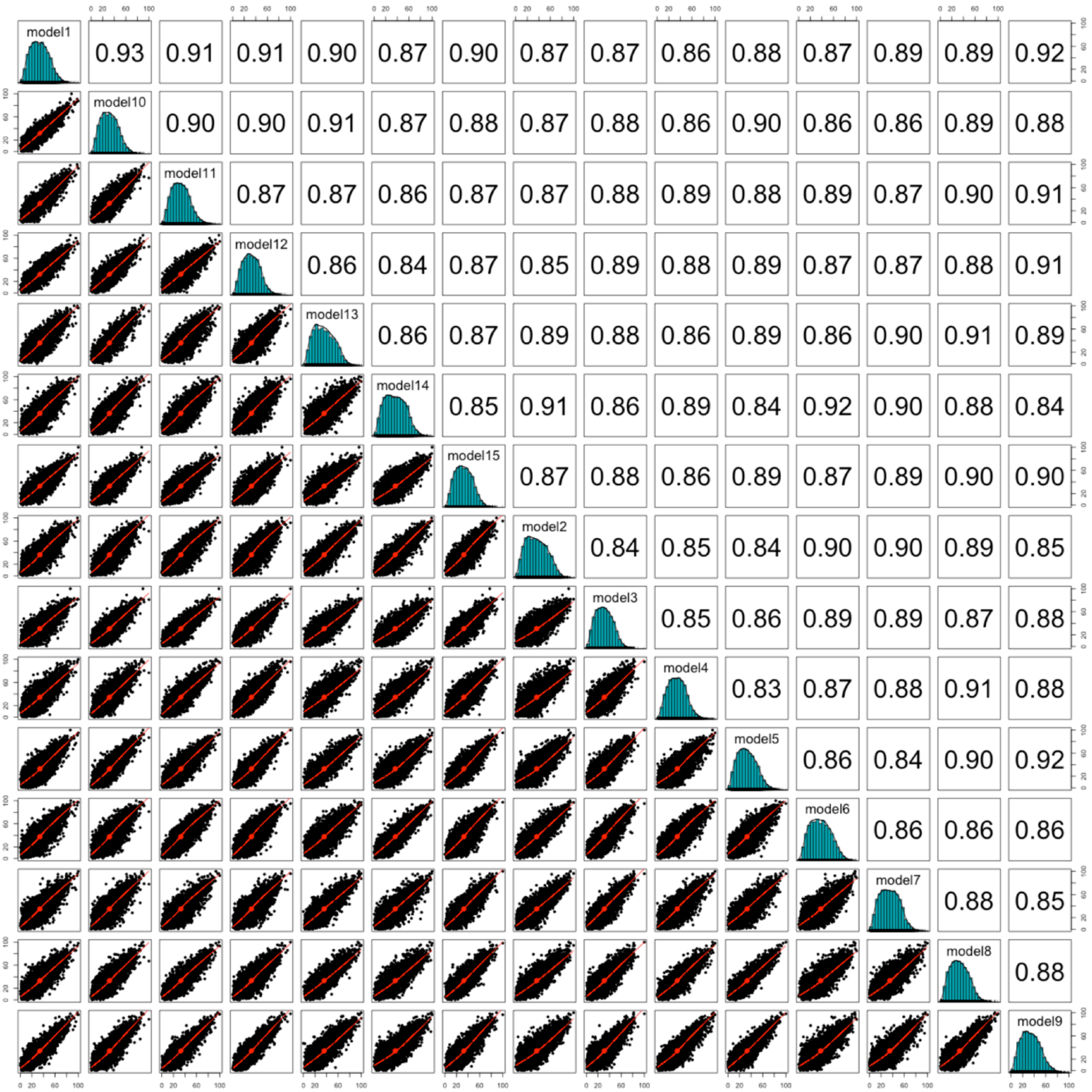

Supplementary Figure 2. Correlation of the variable importance among the different data splits for the transcript-based model.

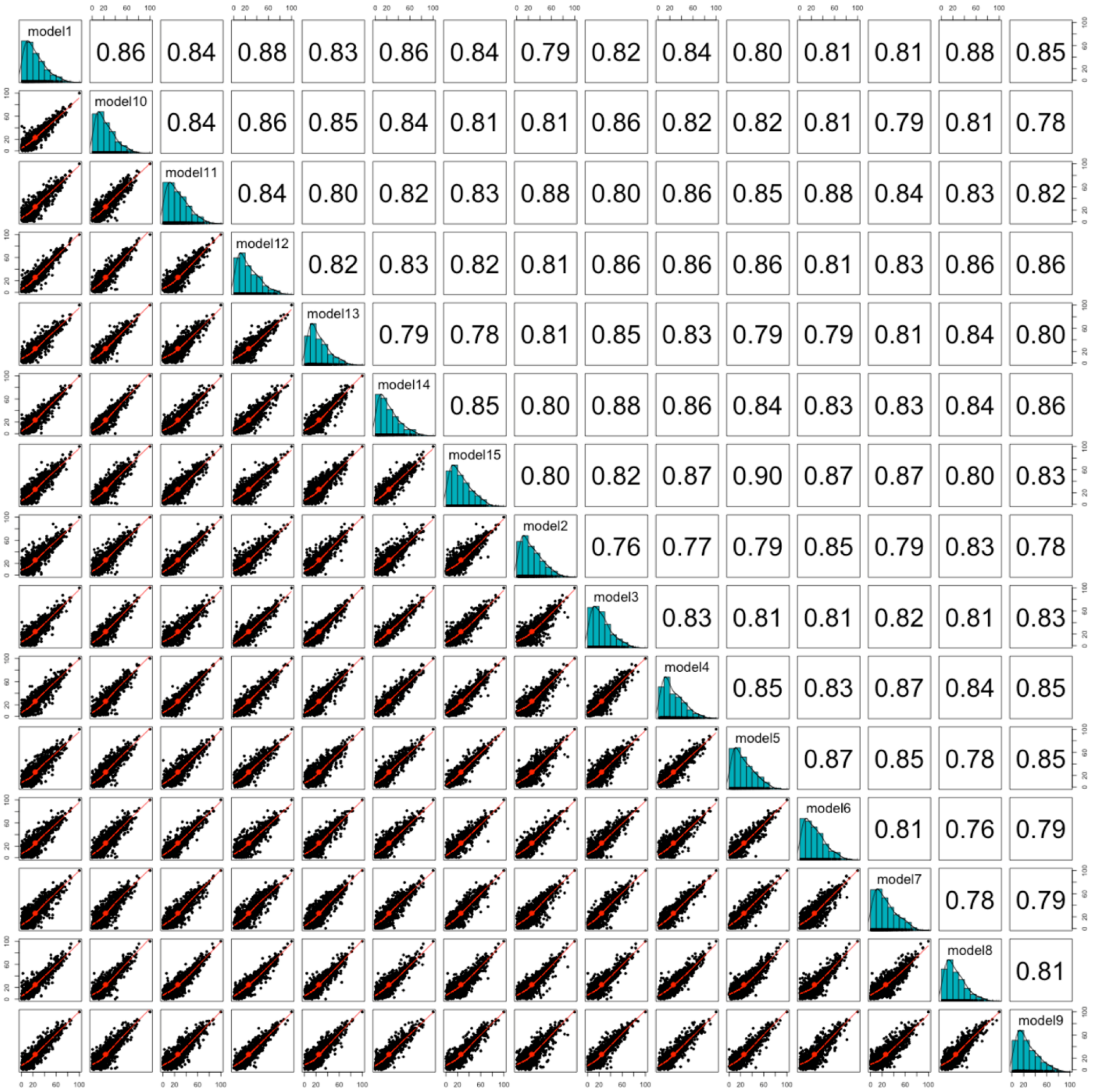

Supplementary Figure 3. Correlation of the variable importance among the different data splits for the protein-based model.

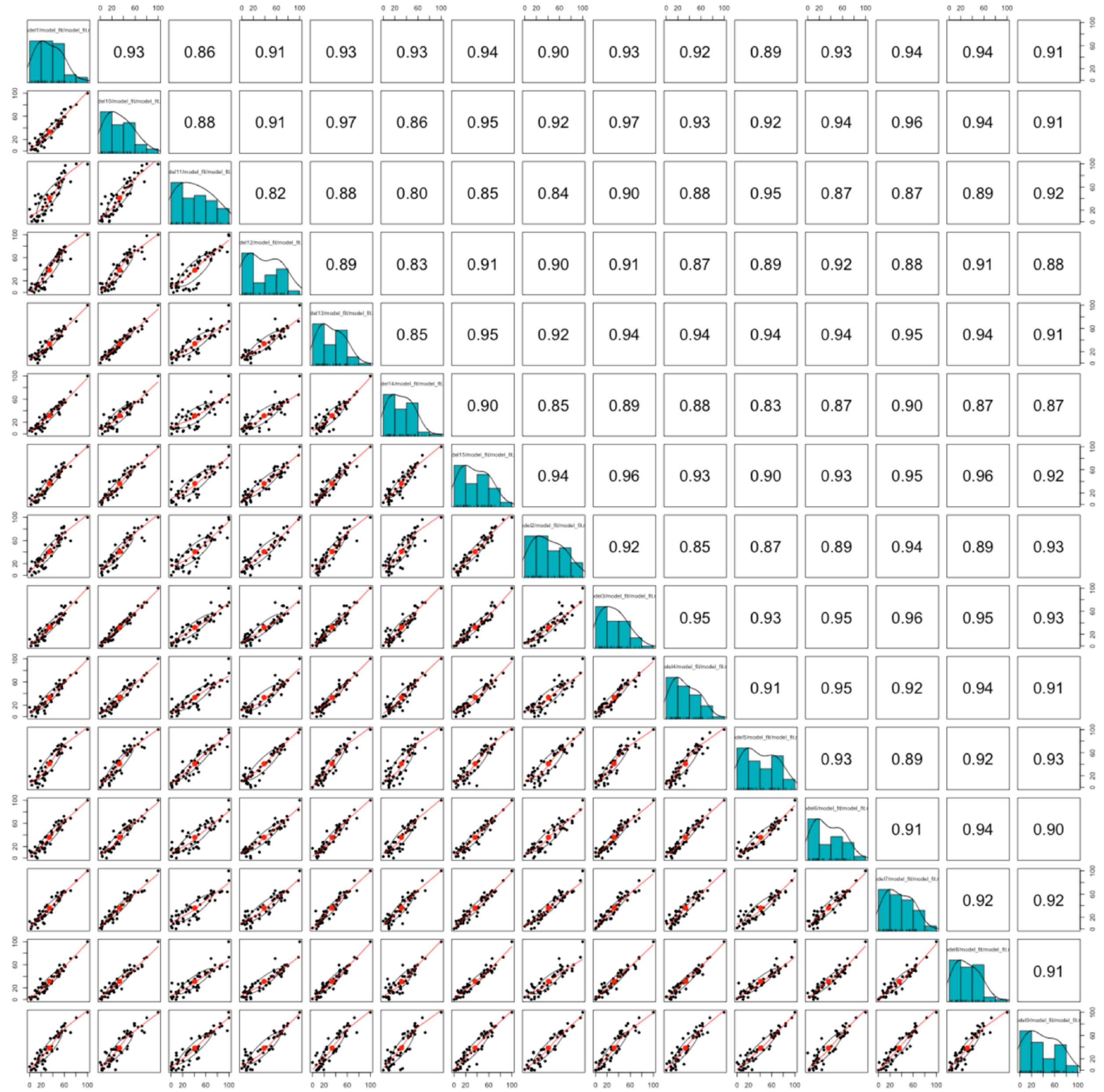

Supplementary Figure 4. Correlation of the variable importance among the different data splits for the cell-based model.

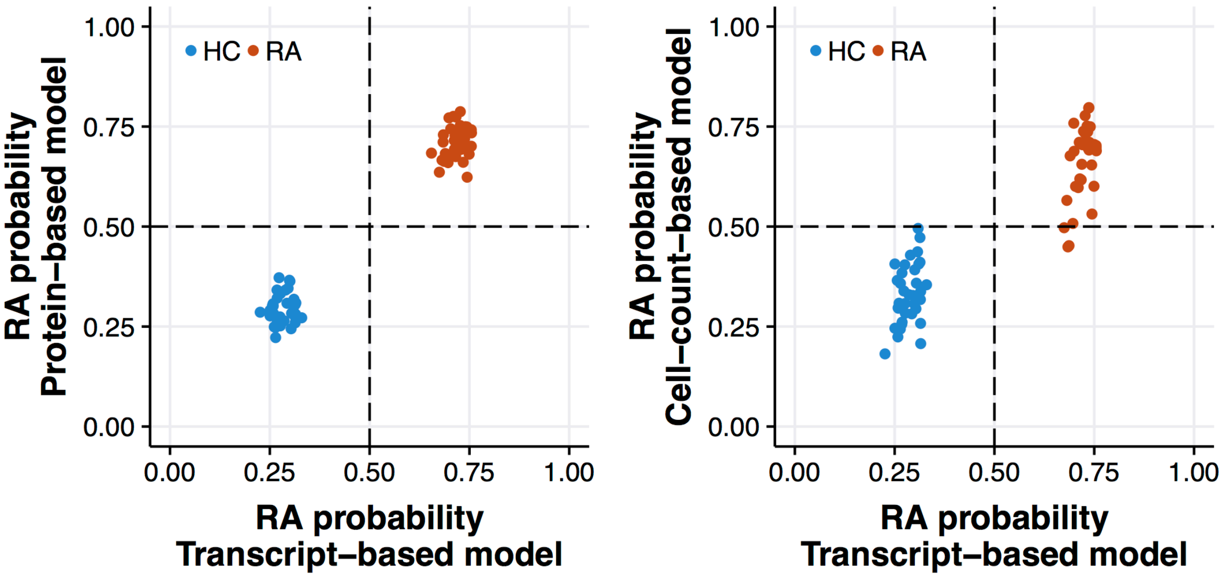

Supplementary Figure 5. RA probability distributions computed via the ensemble diagnostic models. For each data type, the consensus prediction was used as the final output by taking an average of the outputs from the ensemble of 15 PLSR models. These distributions represent predictions against the training data.

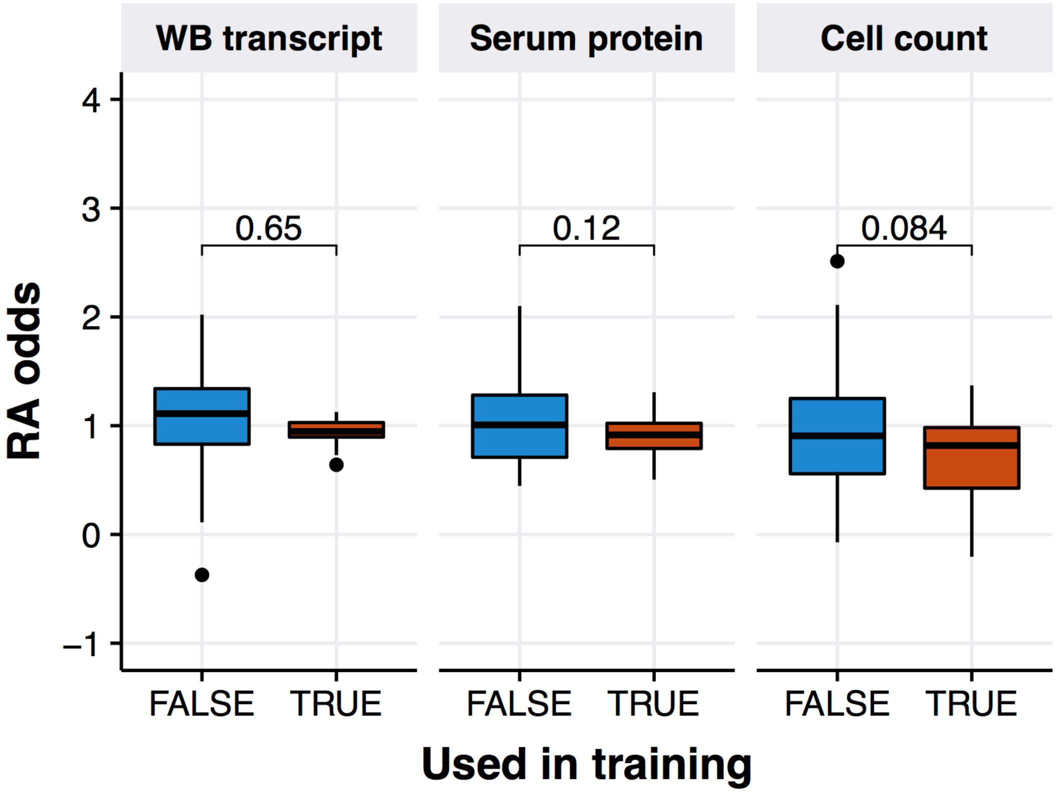

Supplementary Figure 6. Comparison of RA odds between training and test samples. P-values from Welch's t-test are indicated above the boxplots. The upper, center and lower line of the boxplot indicates 75%, 50%, and 25% quantile, respectively. The upper and lower whisker of the boxplot indicates 75% quantile + 1.5 * interquartile range (IQR) and 25% quantile - 1.5 * IQR.

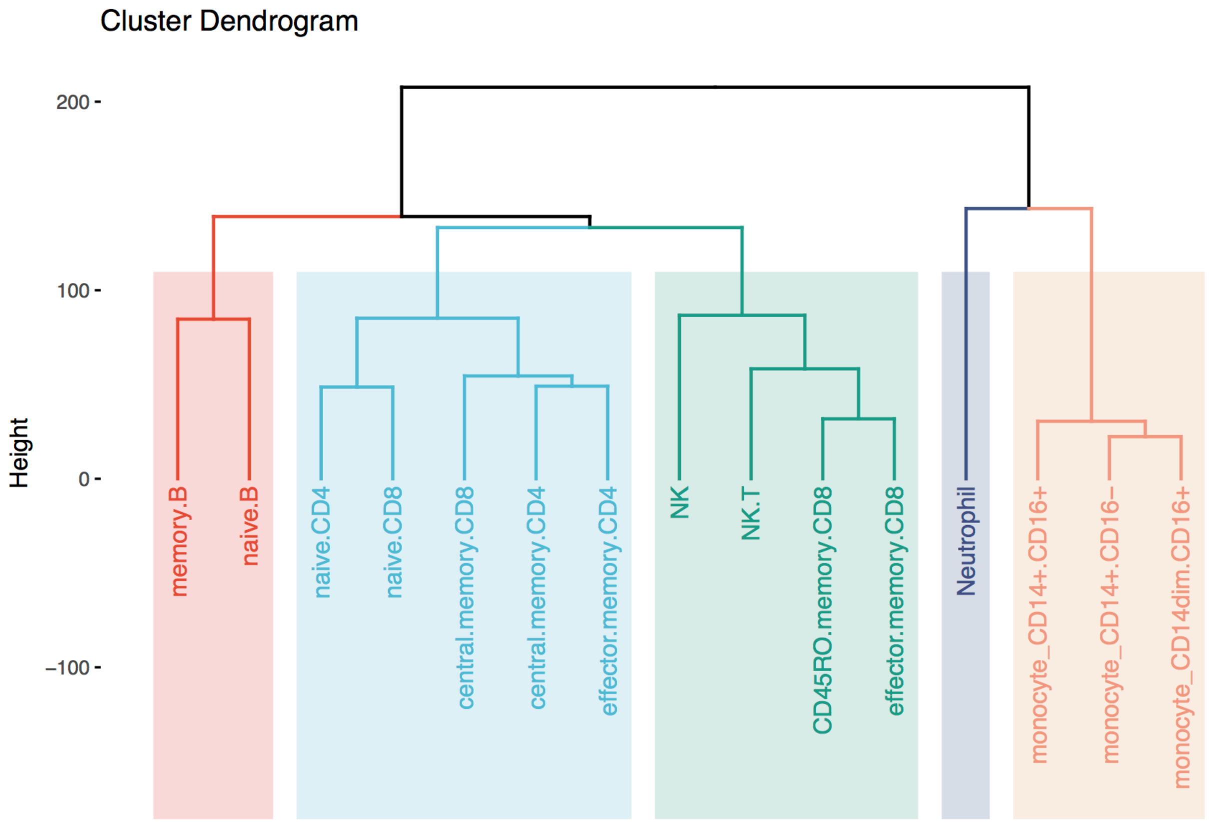

Supplementary Figure 7. Hierarchical clustering of gene expression profiles from 15 immune cells. Expression profiles from 15 immune cells were clustered by hierarchical clustering based on a complete agglomeration with the Euclidean distance.

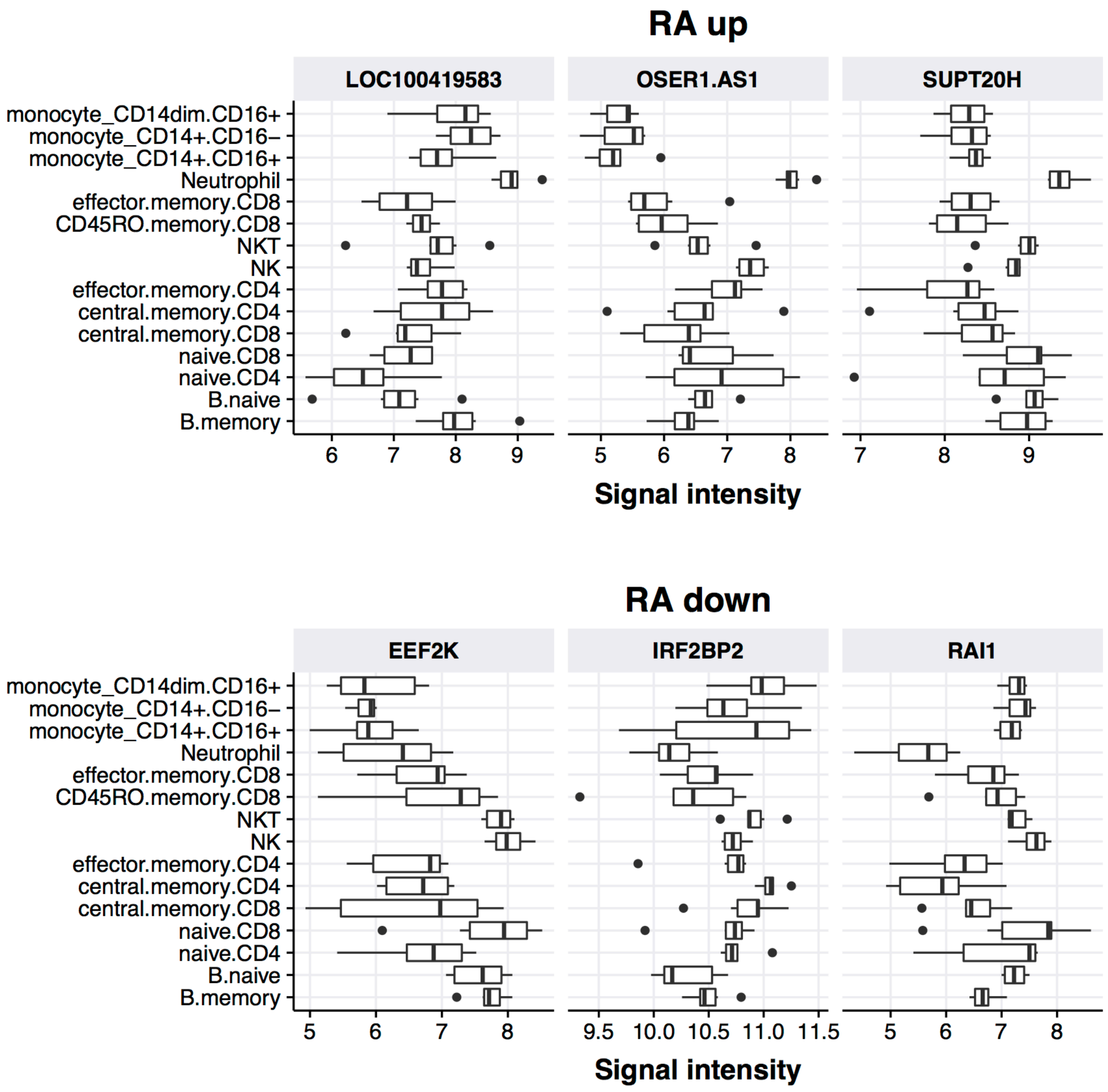

Supplementary Figure 8. Expression profiles of key transcripts in the model across 15 immune cells. The upper, center and lower line of the boxplot indicates 75%, 50%, and 25% quantile, respectively. The upper and lower whisker of the boxplot indicates 75% quantile + 1.5 * interquartile range (IQR) and 25% quantile - 1.5 * IQR.

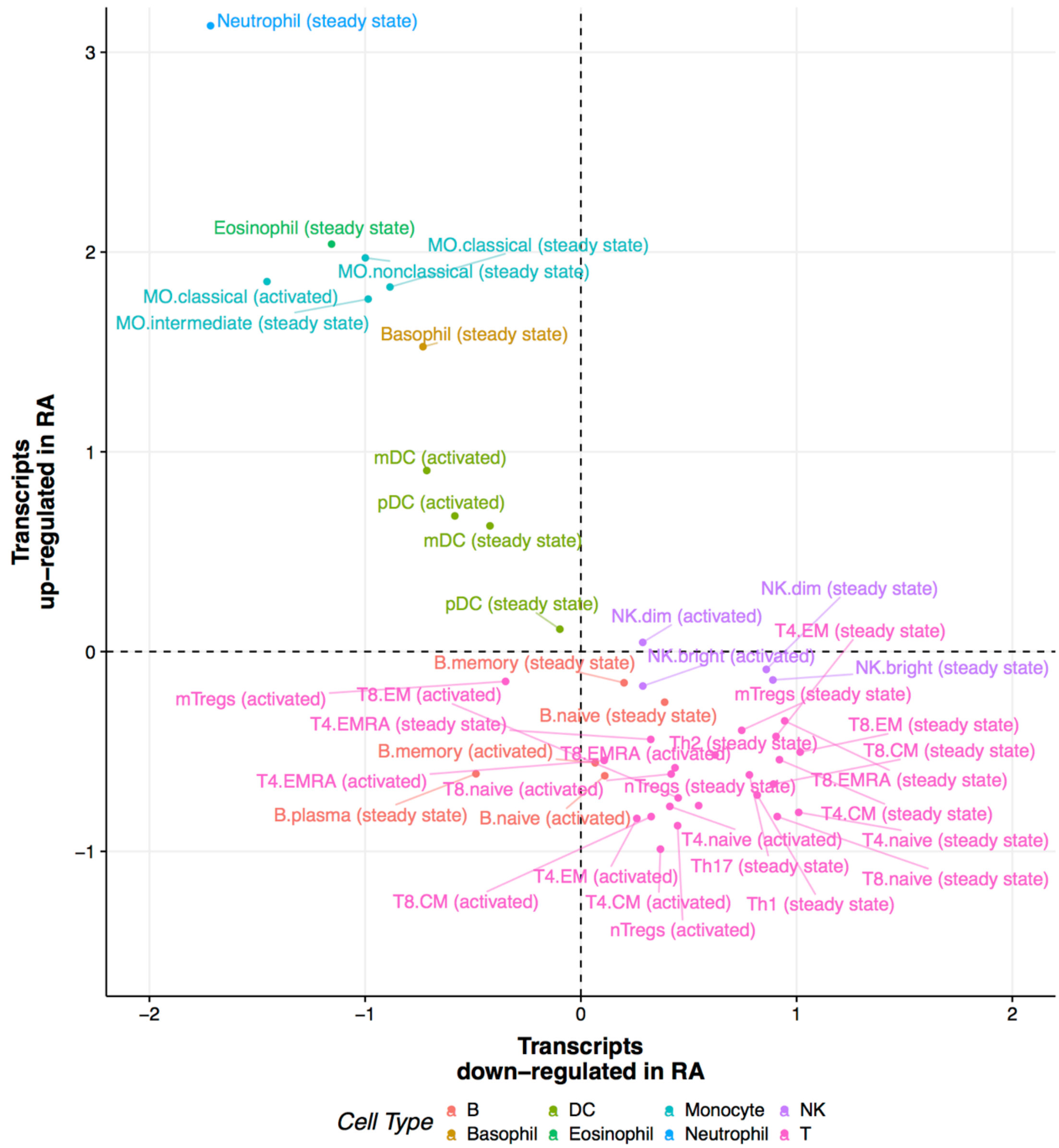

Supplementary Figure 9. Protein expression profiles of important transcripts across 26 immune cells. Meta-expression features for the key upregulated or downregulated transcripts in RA were calculated separately using the ssGSEA method based on the protein expression profiles of 26 immune cells and standardized across immune cells.

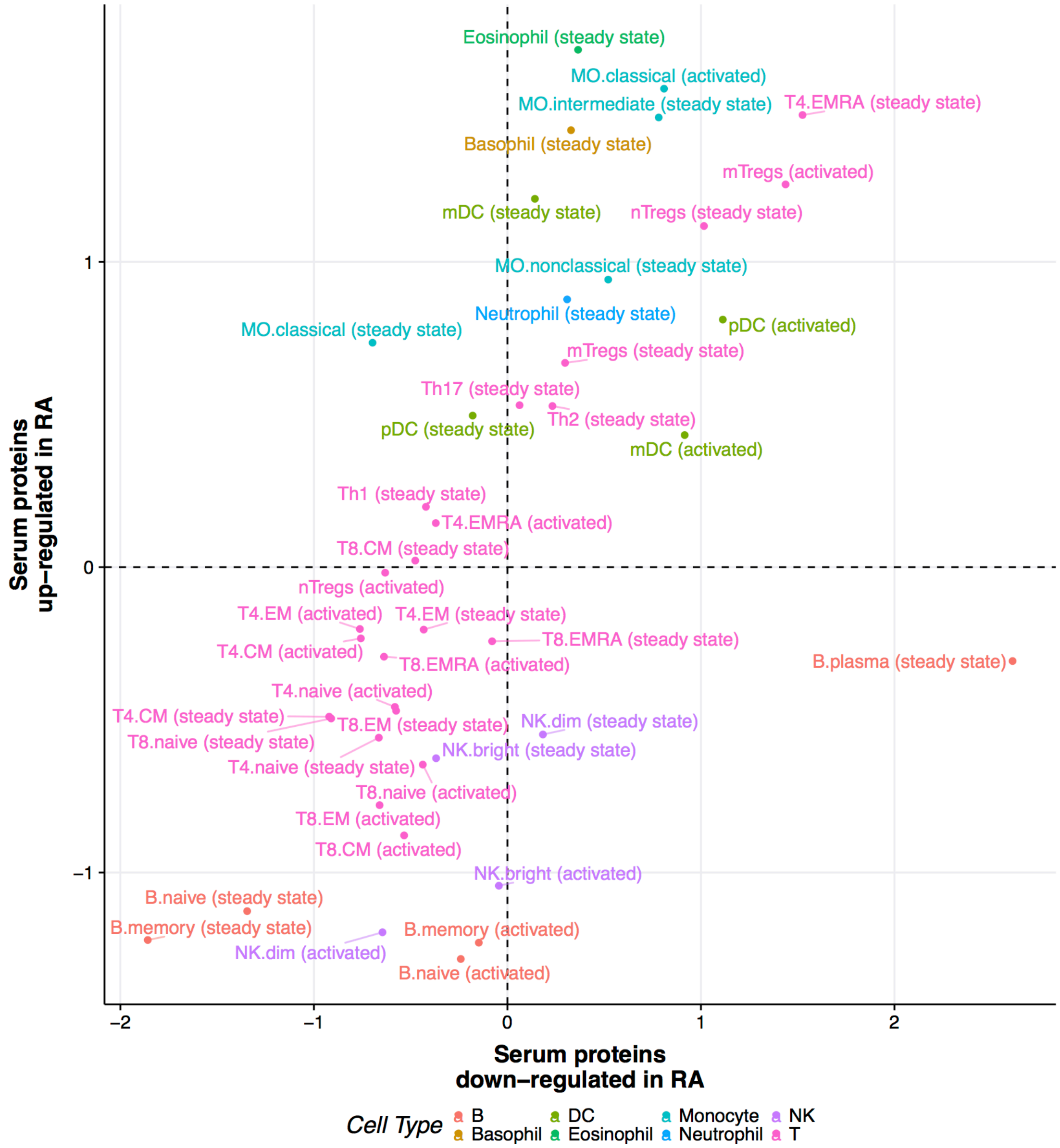

Supplementary Figure 10. Protein expression profiles of key serum proteins across 26 immune cells. Meta-expression features for the key upregulated or downregulated serum proteins in RA were calculated separately using the ssGSEA method based on the protein expression profiles of 26 immune cells and standardized across immune cells.

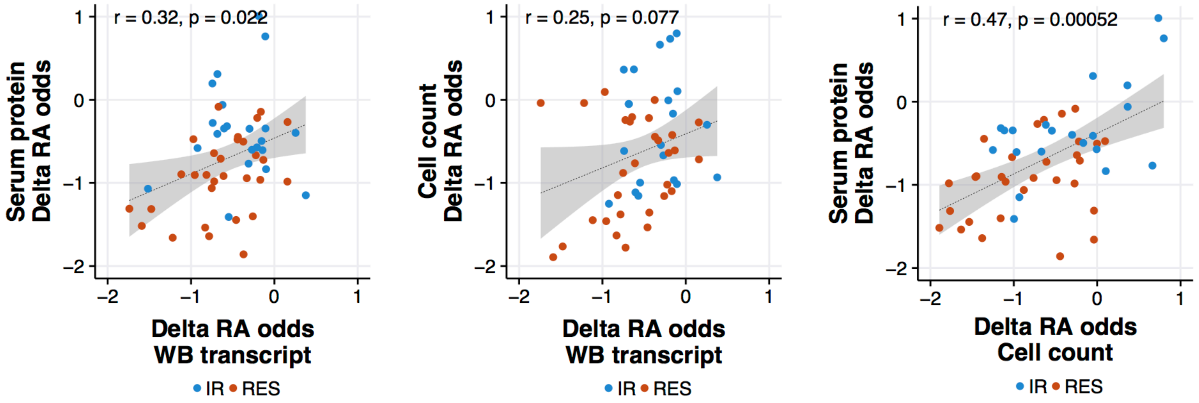

Supplementary Figure 11. Correlation between treatment effects on RA odds based on three molecular classes. The changes in RA odds between 24 and 0 weeks were calculated and compared between molecular classes.

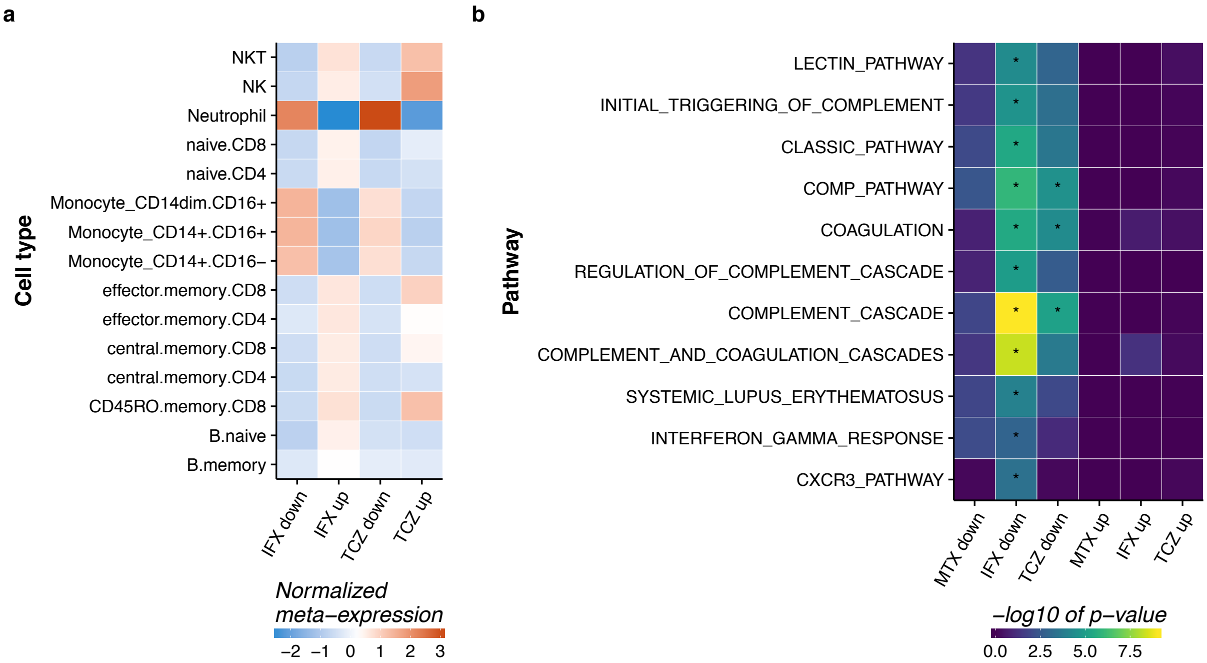

Supplementary Figure 12. Biological annotations for molecular signatures affected by drug treatments. (a) Expression profiles of transcripts affected by drug treatments across 15 immune cells. Meta-expression features for upregulated or downregulated transcripts by drug treatments were calculated separately using the ssGSEA method based on the expression profiles of 15 immune cells and standardized across immune cells. (b) Pathway enrichment analysis for serum proteins affected by drug treatments. The asterisk represents a p-value < 0.05 and FDR < 0.05 based on hypergeometric test.

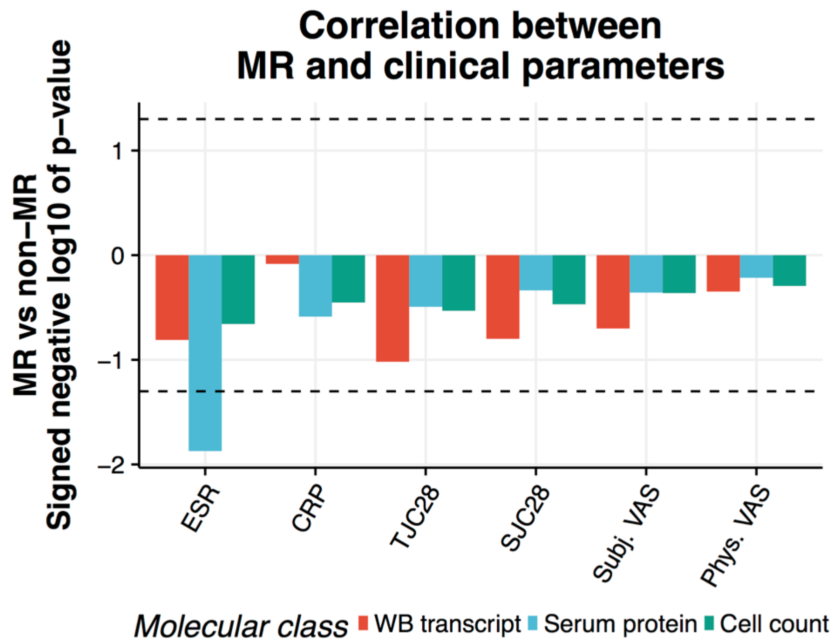

Supplementary Figure 13. Relationship between molecular remission and individual parameters of DAS28-ESR and CDAI at 24 weeks. A dashed line represents a p-value corresponding to 0.05 based on Welch's t-test.

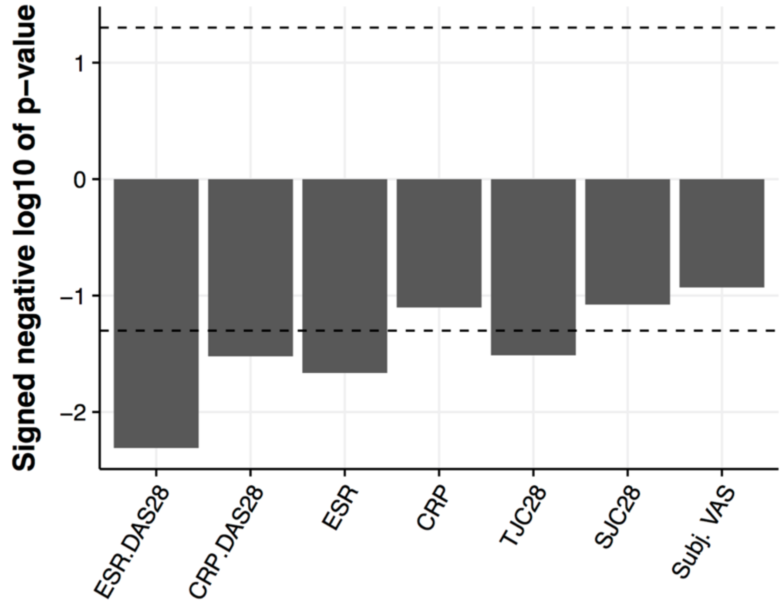

Supplementary Figure 14. Relationship between the number of archived molecular remission classes and individual parameters of DAS28-ESR and CDAI in biologic-treated patients at 90 weeks during the follow-up. A dashed line represents a p-value corresponding to 0.05 based on linear regression.

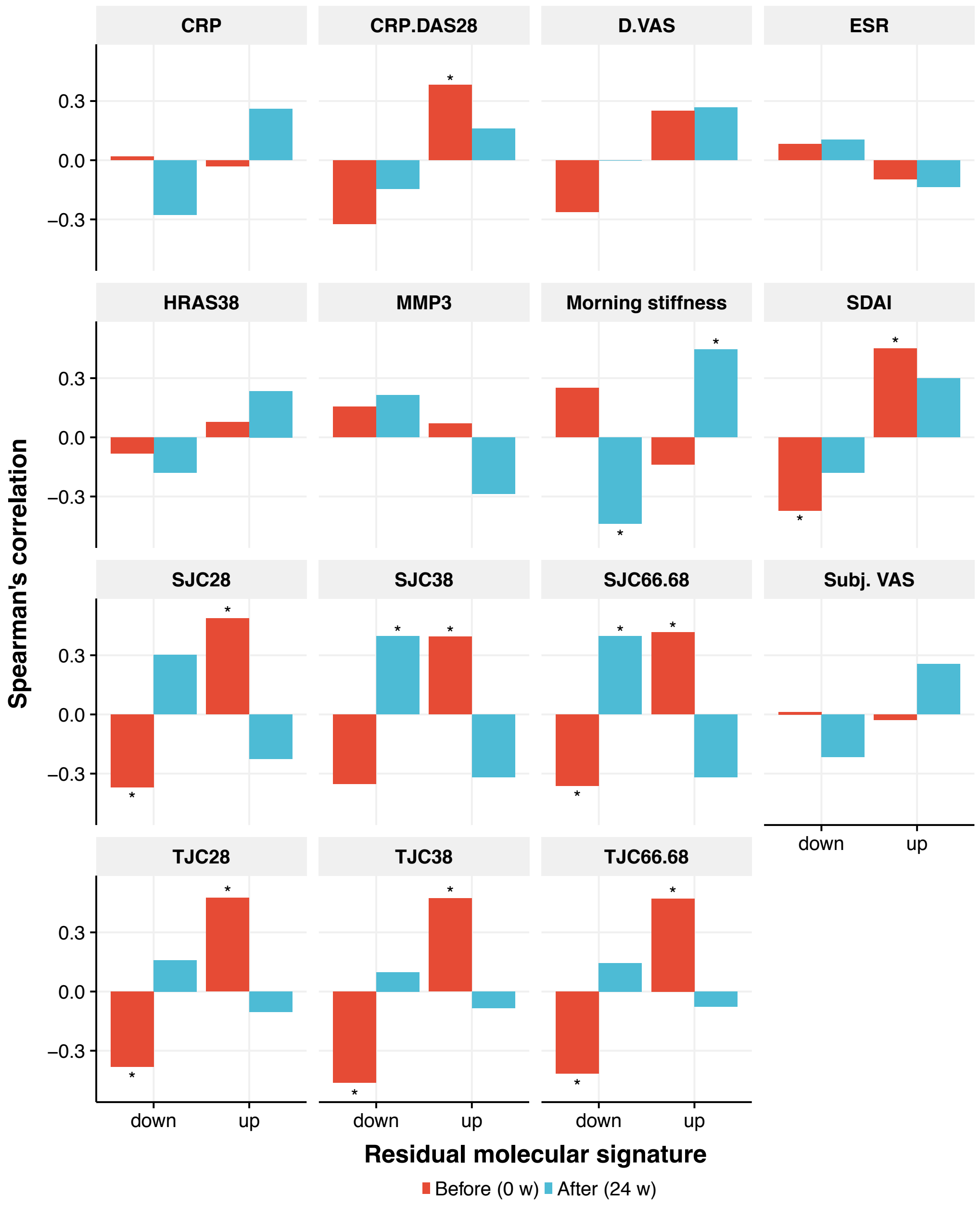

Supplementary Figure 15. Correlation between disease severity indexes and transcriptional RMSs. The levels of meta-features for transcriptional RMSs were contrasted with disease severity indexes before and after treatment (Spearman's correlation; n=30; *p < 0.05). The handy rheumatoid activity score with 38 joints (HRAS38), tenderness and swollen joint counts using 38 or 66/68 joints (TJC38, SJC38, TJC66/68, SJC66/68 values, respectively) were used in this analysis in addition to major disease severity indexes.

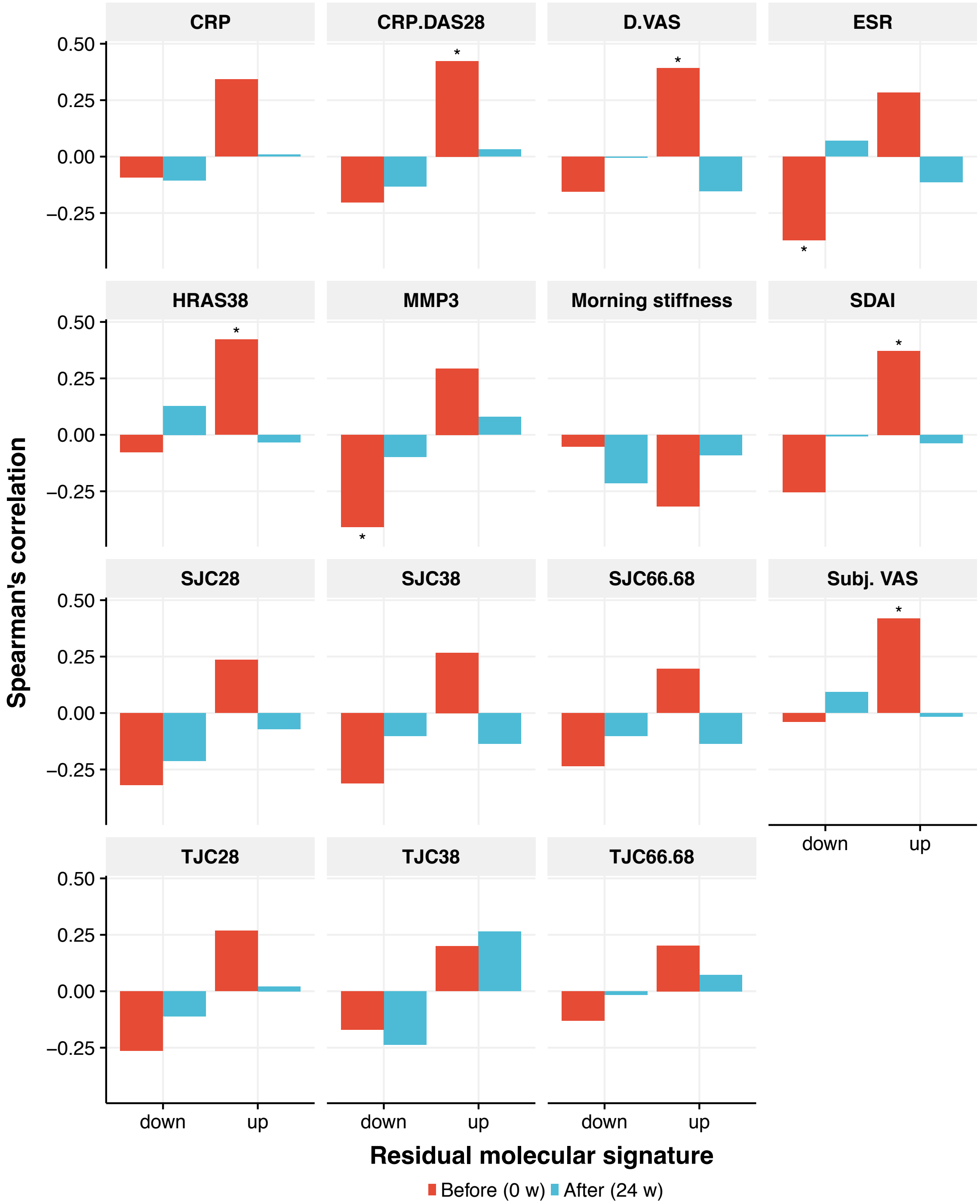

Supplementary Figure 16. Correlation between disease severity indexes and protein RMSs. The levels of meta-features for protein RMSs were contrasted with disease severity indexes before and after treatment (Spearman's correlation; n=30; *p < 0.05).

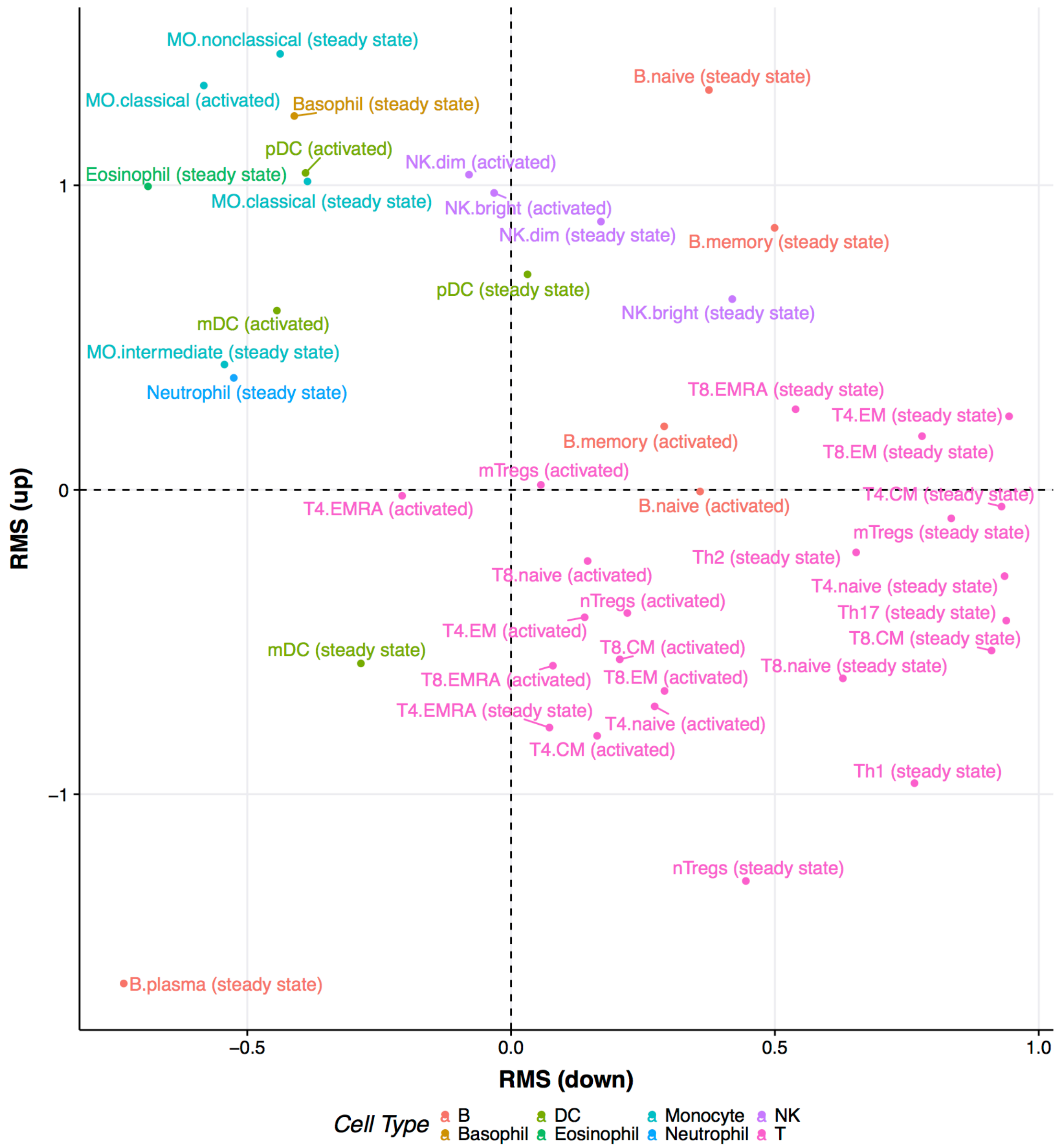

Supplementary Figure 17. Protein expression profiles of transcriptional RMSs across 26 immune cells. Meta-expression features for transcriptional RMSs that were upregulated or downregulated in RA were calculated separately using the ssGSEA method based on the protein expression profiles of 26 immune cells and standardized across immune cells.

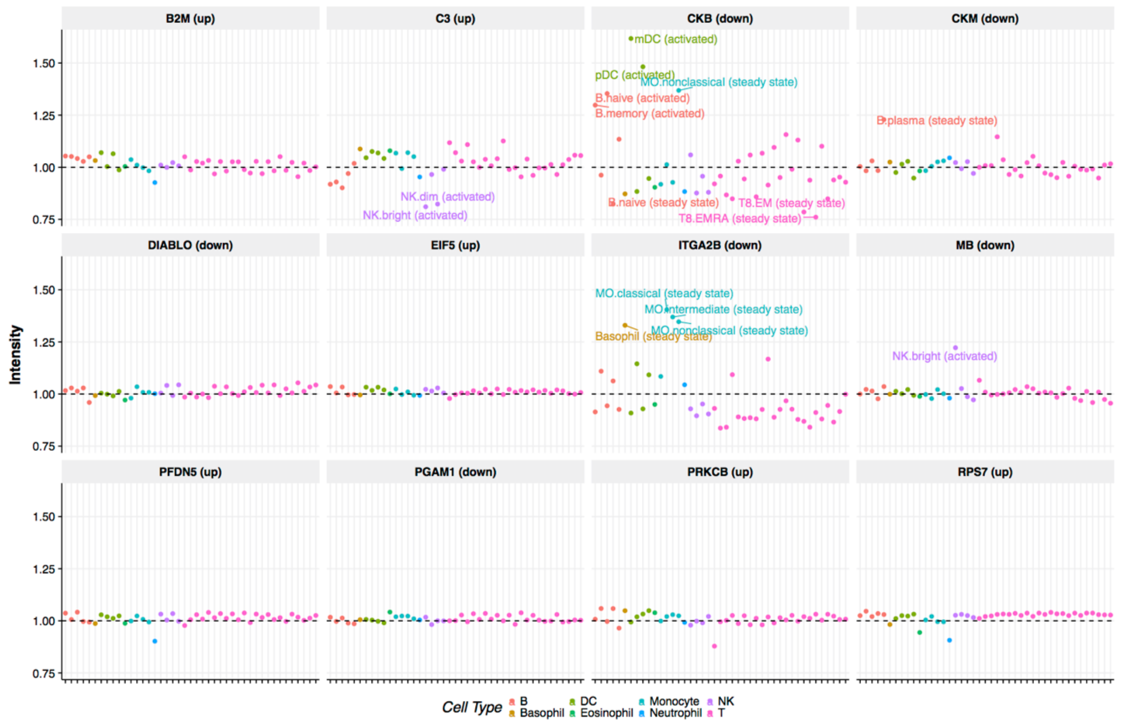

Supplementary Figure 18. Protein expression profiles of protein RMSs across 26 immune cells. The levels of each protein were standardized based on the average of 26 immune cells.

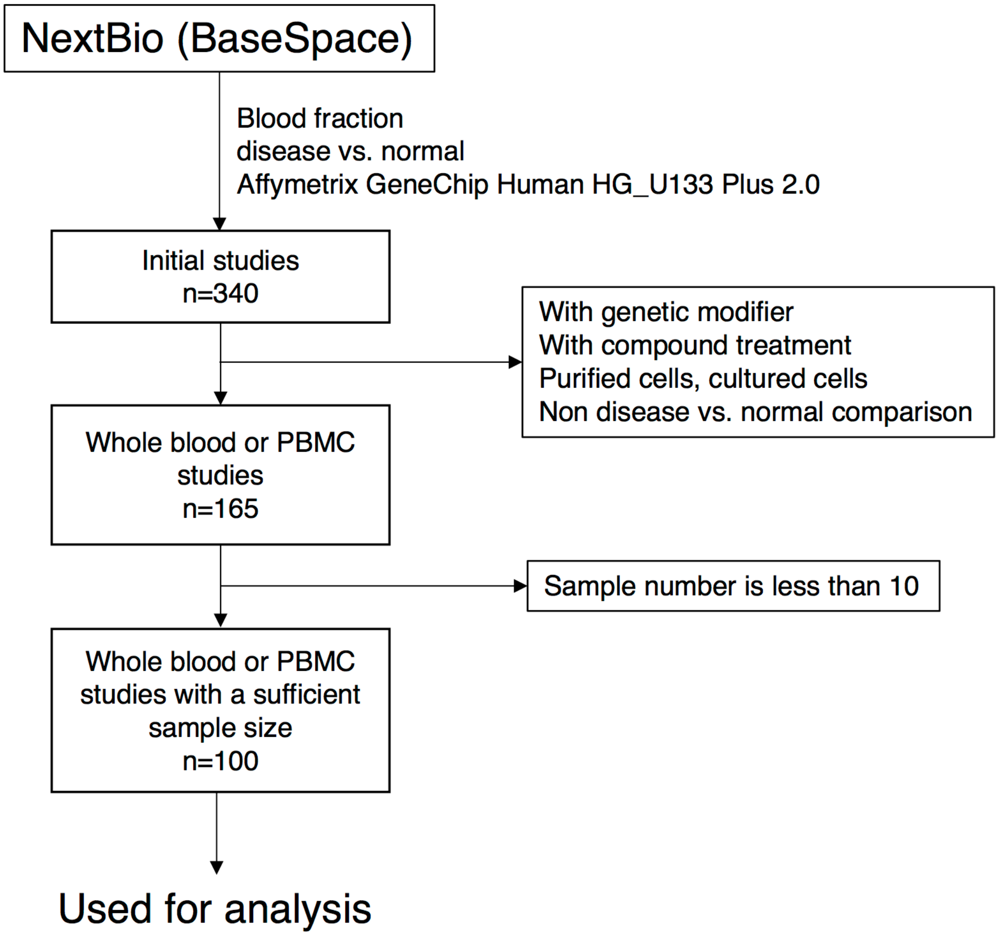

Supplementary Figure 19. Filtering scheme for public transcriptome studies in NextBio.

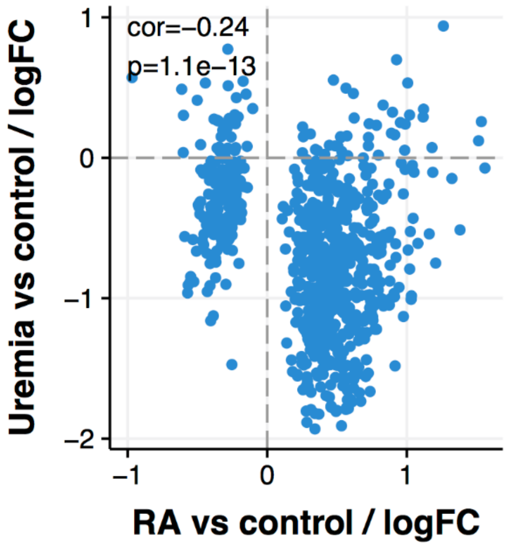

Supplementary Figure 20. Comparison of fold changes for 800 untreatable transcript signatures between RA and other conditions. The fold changes in the 800 untreatable transcripts between patients with uremia (n=75) and controls (n=40) were calculated using the raw data from the uremia study (GSE37171). The fold changes from each study were then compared with those determined for our RA cohort.

	HC	RA	SjS
n	35	45	30
AGE (mean (sd))	41.11 (9.82)	56.33 (15.20)	61.07 (10.80)
GENDER = M (%)	5 (14.3)	7 (15.6)	1 (3.3)
RF = positive (%)		29 (64.4)	
ACPA = positive (%)		29 (64.4)	
CRP [mg/dl] (mean (sd))		1.31 (1.68)	
ESR [mm/hr] (mean (sd))		54.87 (32.33)	
DAS28 CRP (mean (sd))		4.22 (1.21)	
DAS28 ESR (mean (sd))		5.14 (1.22)	
SDAI (mean (sd))		21.77 (14.13)	
CDAI (mean (sd))		20.46 (13.31)	
HAQ-DI (mean (sd))		0.92 (0.70)	
TJC28 (mean (sd))		5.93 (5.52)	
SJC28 (mean (sd))		5.84 (5.79)	
Physician GA, VAS [mm] (mean (sd))		36.42 (21.65)	
Subject GA, VAS [mm] (mean (sd))		50.36 (23.25)	

Supplementary Table 1. Demographics of the unmedicated cohort.

	RA with medication
n	22
AGE (mean (sd))	59.00 (16.83)
GENDER = M (%)	3 (13.6)
RF = positive (%)	17 (85.0)
ACPA = positive (%)	16 (80.0)
CRP [mg/dl] (mean (sd))	2.16 (1.81)
ESR [mm/hr] (mean (sd))	68.95 (37.35)
DAS28 CRP (mean (sd))	5.25 (1.24)
DAS28 ESR (mean (sd))	6.13 (1.29)
SDAI (mean (sd))	32.24 (17.75)
CDAI (mean (sd))	30.08 (16.61)
HAQ-DI (mean (sd))	1.45 (0.78)
TJC28 (mean (sd))	9.41 (6.86)
SJC28 (mean (sd))	9.68 (7.01)
Physician GA, VAS [mm] (mean (sd))	47.73 (23.57)
Subject GA, VAS [mm] (mean (sd))	62.14 (22.33)

Supplementary Table 2. Demographics of 22 patients with RA receiving medication.

ID	Source	Description	p.Val	FDR	Direction	OverlapGenes
REACTOME_COMPLEMENT_CASCADE	c2.cp.v5.1.entrez	Genes involved in Complement cascade	1.31173E-07	0.000284383	UP	C9,C3,CRP,C1S,C5,CFH,CFI,CFB,C4A
PID_TELOMERASE_PATHWAY	c2.cp.v5.1.entrez	Regulation of Telomerase	2.72212E-05	0.019671881	UP	MAPK1,MAPK3,XRCC6,HSP90AA1,IL2
REACTOME_REGULATION_OF_COMPLEMENT_CASCADE	c2.cp.v5.1.entrez	Genes involved in Regulation of Complement cascade	2.72212E-05	0.019671881	UP	C3,CFH,CFI,CFB,C4A
REACTOME_INITIAL_TRIGGERING_OF_COMPLEMENT	c2.cp.v5.1.entrez	Genes involved in Initial triggering of complement	5.85266E-05	0.026813385	UP	C3,CRP,C1S,CFB,C4A
BIOCARTA_COMP_PATHWAY	c2.cp.v5.1.entrez	Complement Pathway	6.1839E-05	0.026813385	UP	C9,C3,C1S,C5,CFB,C4A
PID_CXCR3_PATHWAY	c2.cp.v5.1.entrez	CXCR3-mediated signaling events	9.84356E-05	0.035568066	UP	MAPK14,MAPK1,MAP2K1,MAPK3,CXCL13,CXCL11
HALLMARK_COAGULATION	h.all.v5.1.entrez	Genes encoding components of blood coagulation system; also up-regulated in platelets.	0.000149765	0.046384452	UP	C1S,CFB,CFI,C3,F9,OLR1,FGA,MMP3,CFH,C9,LTA4H,ANXA1

Supplementary Table 3. Biological concepts enriched with important variables in the protein-based model.

	IFX(IR)	IFX(R)	MTX(IR)	MTX(R)	TCZ(IR)	TCZ(R)
n	8	10	11	10	3	10
AGE (mean (sd))	50.75 (18.88)	51.10 (16.91)	66.91 (8.34)	58.70 (10.63)	68.67 (11.72)	58.00 (14.89)
GENDER = M (%)	1 (12.5)	0 (0.0)	3 (27.3)	2 (20.0)	1 (33.3)	0 (0.0)
RF = positive (%)	6 (85.7)	6 (75.0)	5 (45.5)	7 (70.0)	2 (100.0)	7 (87.5)
ACPA = positive (%)	6 (85.7)	6 (75.0)	4 (36.4)	7 (70.0)	2 (100.0)	6 (75.0)
CRP [mg/dl] (mean (sd))	1.33 (1.03)	1.49 (1.05)	2.01 (2.51)	0.99 (1.04)	4.19 (2.92)	1.70 (1.92)
ESR [mm/hr] (mean (sd))	48.25 (34.87)	60.10 (31.24)	56.00 (38.31)	50.30 (32.40)	103.00 (50.86)	67.80 (34.05)
DAS28 CRP (mean (sd))	4.06 (1.18)	5.46 (1.21)	4.24 (0.97)	4.15 (0.75)	5.80 (2.60)	4.86 (1.38)
DAS28 ESR (mean (sd))	4.81 (1.34)	6.35 (1.25)	5.04 (0.96)	4.97 (0.76)	6.75 (2.65)	5.88 (1.25)
SDAI (mean (sd))	19.71 (10.96)	35.50 (18.90)	20.84 (11.29)	18.96 (7.47)	45.46 (30.77)	27.07 (17.80)
CDAI (mean (sd))	18.38 (10.83)	34.01 (18.06)	18.84 (9.49)	17.97 (7.42)	41.27 (27.86)	25.37 (16.76)
HAQ-DI (mean (sd))	0.72 (0.46)	1.50 (0.43)	1.09 (0.77)	0.85 (0.61)	1.92 (1.04)	1.49 (0.88)
TJC28 (mean (sd))	4.62 (4.31)	11.40 (7.81)	4.09 (3.30)	5.20 (3.46)	13.33 (11.55)	7.80 (6.55)
SJC28 (mean (sd))	4.75 (4.37)	11.30 (8.00)	5.27 (5.61)	5.00 (3.30)	14.00 (12.12)	7.70 (6.46)
PAIN.VAS (mean (sd))	52.25 (17.56)	67.56 (19.60)	59.73 (20.60)	41.10 (25.24)	76.00 (9.17)	64.20 (28.08)
Physician GA, VAS [mm] (mean (sd))	37.62 (18.15)	48.30 (22.17)	34.00 (23.82)	29.40 (11.50)	63.33 (41.74)	37.30 (24.63)
Subject GA, VAS [mm] (mean (sd))	52.38 (15.26)	64.80 (18.84)	60.73 (21.23)	48.30 (15.53)	76.00 (9.17)	61.40 (27.73)

Supplementary Table 4. Demographics of the drug response cohort.

	HC:B	HC:CD4T	RA:CD4T	HC:CD8T	RA:CD8T	HC:Mono	RA:Mono
n	6	6	6	6	6	6	6
AGE (mean (sd))	43.17 (12.22)	43.33 (5.72)	50.33 (21.81)	43.83 (6.55)	46.50 (16.50)	43.67 (6.31)	59.00 (19.15)
GENDER = M (%)	0 (0.0)	0 (0.0)	2 (33.3)	0 (0.0)	2 (33.3)	0 (0.0)	3 (50.0)
RF = positive (%)			6 (100.0)		5 (83.3)		4 (66.7)
ACPA = positive (%)			5 (83.3)		4 (66.7)		3 (50.0)
CRP [mg/dl] (mean (sd))			2.15 (2.05)		1.45 (1.12)		2.81 (2.02)
ESR [mm/hr] (mean (sd))			58.67 (47.83)		54.67 (41.91)		83.67 (46.28)
DAS28 CRP (mean (sd))			5.32 (1.11)		4.86 (0.69)		5.06 (1.22)
DAS28 ESR (mean (sd))			6.01 (1.48)		5.60 (1.07)		5.92 (1.50)
SDAI (mean (sd))			32.77 (16.93)		25.12 (8.82)		28.76 (19.03)
CDAI (mean (sd))			30.62 (15.61)		23.67 (9.13)		25.95 (18.08)
HAQ-DI (mean (sd))			1.15 (0.90)		0.75 (0.47)		1.02 (0.95)
TJC28 (mean (sd))			9.83 (5.53)		7.33 (2.66)		8.17 (6.40)
SJC28 (mean (sd))			9.67 (6.25)		6.50 (3.62)		7.50 (7.66)
PAIN.VAS (mean (sd))			58.33 (23.64)		55.00 (23.42)		52.67 (20.20)
Physician GA, VAS [mm] (mean (sd))			52.50 (25.64)		41.50 (13.50)		46.83 (29.30)
Subject GA, VAS [mm] (mean (sd))			58.67 (21.18)		56.83 (20.68)		56.00 (17.61)

	HC:Neu	RA:Neu	HC:NK	RA:NK	HC:NKT	RA:NKT
n	6	9	6	7	6	7
AGE (mean (sd))	46.00 (2.61)	61.11 (11.27)	43.83 (6.55)	52.14 (18.84)	43.83 (6.55)	55.71 (22.01)
GENDER = M (%)		2 (22.2)		2 (28.6)		2 (28.6)
RF = positive (%)		5 (55.6)		5 (71.4)		5 (71.4)
ACPA = positive (%)		5 (55.6)		4 (57.1)		5 (71.4)
CRP [mg/dl] (mean (sd))		1.64 (1.38)		1.88 (1.49)		2.18 (2.00)
ESR [mm/hr] (mean (sd))		69.33 (40.97)		72.00 (42.88)		87.71 (37.95)
DAS28 CRP (mean (sd))		4.65 (0.80)		4.81 (0.68)		5.19 (1.09)
DAS28 ESR (mean (sd))		5.57 (1.10)		5.70 (1.03)		6.27 (1.11)
SDAI (mean (sd))		22.47 (9.20)		24.14 (8.93)		30.47 (16.84)
CDAI (mean (sd))		20.83 (9.60)		22.26 (9.67)		28.29 (15.86)
HAQ-DI (mean (sd))		1.11 (0.80)		0.75 (0.43)		1.12 (0.79)
TJC28 (mean (sd))		6.11 (4.62)		6.43 (2.76)		8.57 (5.71)
SJC28 (mean (sd))		4.11 (3.44)		5.86 (3.98)		8.29 (6.82)
PAIN.VAS (mean (sd))		61.56 (22.06)		58.57 (23.00)		63.57 (20.14)
Physician GA, VAS [mm] (mean (sd))		39.33 (15.02)		39.86 (14.97)		49.29 (25.95)
Subject GA, VAS [mm] (mean (sd))		66.78 (19.30)		59.86 (18.81)		65.00 (14.31)

Supplementary Table 5. Demographics of the immune subset cohort.
