# Supplementary material for: Multi-omics monitoring of drug response in rheumatoid arthritis in pursuit of molecular remission
Source: Nat Commun. 2018 Jul 16;9:2755. doi: 10.1038/s41467-018-05044-4 (PMC6048065; doi:10.1038/s41467-018-05044-4)
Supplement: Supplementary file 3 — Description of Additional Supplementary Files [file 41467_2018_5044_MOESM3_ESM.pdf]

Description of Additional Supplementary Files

File Name: Supplementary Data 1
Description: Variable importance statistics from PLSR models of the mRNA microarray, protein array and cell abundance.

File Name: Supplementary Data 2
Description: Variables affected by MTX, IFX and TCZ.

File Name: Supplementary Data 3
Description: Untreatable variables for MTX, IFX and TCZ of the mRNA microarray, and protein array.

File Name: Supplementary Data 4
Description: Disease-wide profile of transcriptional RMSs.

File Name: Supplementary Data 5
Description: Samples used in omics profiling.
